# Telerehabilitation Delivery of Multi-Modality Aphasia Therapy (M-MAT Tele): A Pilot Feasibility Trial

**DOI:** 10.63144/ijt.2025.6727

**Published:** 2025-12-12

**Authors:** Vicky Aston, Emily Eley, Dana Wong, Annie J Hill, Marcella Carragher, Miranda L Rose, Rachelle Pitt, John E Pierce

**Affiliations:** 1 Peninsula Health, Melbourne, Australia; 2 Centre of Research Excellence in Aphasia Recovery and Rehabilitation, La Trobe University, Melbourne, Australia; 3 School of Allied Health Human Services and Sport, La Trobe University, Melbourne, Australia; 4 School of Psychology and Public Health, La Trobe University, Bundoora, Australia; 5 Queensland Health, Queensland, Australia; 6 School of Health and Rehabilitation Sciences, The University of Queensland, St. Lucia, Australia

**Keywords:** Aphasia, Aphasia Therapy, Group Therapy, Multi-Modality, Telerehabilitation

## Abstract

Multi-Modality Aphasia Therapy (M-MAT) is a cost-effective group intervention for post stroke aphasia. M-MAT was recently adapted for telerehabilitation but has not yet been tested. This pilot study aimed to investigate the feasibility, acceptability, and preliminary signs of efficacy, providing 30 hours of M-MAT Tele to three groups of three people with chronic aphasia. Participants were grouped according to aphasia severity. Clinical outcomes were assessed at three timepoints. Feasibility and acceptability were assessed through a range of trial measures including recruitment, adherence, treatment fidelity and overall participant ratings. Recruitment targets were achieved, with nine participants receiving a median 24.4h of the prescribed dose. Therapy integrity and adherence were high (94%), effect sizes favoured positive clinical change, and acceptability was strong based on participant and therapist feedback. Minor protocol/software changes were recommended. This pilot study showed that M-MAT Tele was acceptable and feasible to participants and therapists, with positive preliminary outcomes.

Globally, there are 10 million new stroke cases each year and of these, over 3 million experience aphasia (Benjamin et al., 2017). Aphasia is an acquired communication disability that can result in impairments in spoken language, comprehension, reading and writing and often persists long term (Flowers et al., 2016). Aphasia has far-reaching negative effects. Interpersonal relationships and interactions with friends and family members are transformed following aphasia onset, as aphasia impacts the opportunities and quality of communicative exchanges (Davidson et al., 2008). Individuals with aphasia experience amongst the lowest health-related quality of life of a range of health conditions (Lam & Wodchis, 2010). Depression rates are estimated to be 7.4 times higher in this population when compared to other adults without aphasia (Zanella et al., 2023). Effective interventions for aphasia are therefore critical.

Research supports the effectiveness of aphasia interventions in improving functional communication, reading, writing, and expressive language (Brady et al., 2016). High intensity, high dose treatment may be more beneficial (Hilari et al., 2024); however, this is not acceptable or accessible to all (Brady et al., 2016). Accessing aphasia rehabilitation is known to be problematic for many due to funding limitations and a lack of services, especially for those in regional and rural areas (Pitt et al., 2017; Teti et al., 2023).

Telerehabilitation is a service model that has had significant growth in its implementation since the COVID-19 pandemic due to the limitations on in-person services (Hilari et al., 2024). For many, telerehabilitation offers a potential solution to access issues as it reduces or eliminates travel for clinicians and clients and provides options for individuals who reside in rural areas (Pitt et al, 2017). Telerehabilitation in stroke has been shown to be effective for a range of outcomes and is generally acceptable to stroke survivors, provided there is appropriate training and technical infrastructure (Stephenson et al., 2022). For stroke survivors with aphasia, there is mounting evidence that telerehabilitation is both feasible and effective (Øra et al., 2020; Vuong et al., 2024; Woolf et al., 2016). Provision of aphasia group therapy via telerehabilitation has also shown significant improvements in language functioning and quality of life (Pitt et al., 2019).

Multi-Modality Aphasia Therapy (M-MAT) is an evidence-based aphasia intervention that may be suitable for delivery over telerehabilitation. M-MAT is traditionally delivered as an intensive, in-person treatment delivered within small groups of 2–3 people with aphasia. M-MAT focuses on improving an individual’s verbal communication at word through to sentence level, using gesture, writing, reading and drawing to facilitate word retrieval. Recent evidence from a rigorous randomised controlled trial has shown that M-MAT is an effective aphasia intervention, with significant improvements in word retrieval, functional communication, and quality of life (Rose et al., 2022). Additional trials have contributed further evidence of the effectiveness of M-MAT (Low et al., 2025; Pierce et al., 2023). M-MAT appears to remain efficacious at a lower intensity of 30 hours over five weeks (compared to 30 hours over two weeks) (Pierce et al., 2023). Providing M-MAT via telerehabilitation may increase access to this effective intervention.

M-MAT has been adapted for telerehabilitation administration (*M-MAT Tele*) through a co-design process involving both people with aphasia and practising clinicians (Pierce et al., 2024) guided by the Human Centred Design Framework (BSI, 2010). However, the adaptation has not yet been tested in people with aphasia outside of the co-design process. Ultimately, we hypothesise that M-MAT Tele may be as effective as the in-person format in improving word retrieval, functional communication, and communicative quality of life.

The primary aim of the current study was to evaluate its feasibility and acceptability, and to identify any necessary refinements to the protocol or software prior to future trials. Specifically, we examined the feasibility and acceptability of M-MAT Tele as an intervention, as well as the feasibility and acceptability of a trial of M-MAT Tele. We also explored a range of potential clinical trial outcomes for preliminary signals of efficacy and selection of outcomes for a larger trial.

## Method

### Study Design

This study was a pre/post, feasibility study (Phase I) which aimed to provide 30 hours of M-MAT over 5 weeks via telerehabilitation to three groups of people with chronic aphasia. A target sample size of nine was selected for this initial feasibility study based on practical considerations including resources and study timeline (Teresi et al., 2022) and enabled the creation of three treatment groups. Groups were planned to run sequentially, with regular opportunities for participant feedback leading to iterative software updates either during or following the intervention phase. The trial was coordinated from the Centre of Research Excellence in Aphasia Recovery and Rehabilitation (Aphasia CRE) at La Trobe University in Melbourne and ethical approval was received from the La Trobe University Human Research Ethics Committee (HEC22264). Results are reported according to the CONSORT pilot and feasibility checklist.

### Participants

**Figure 1:**
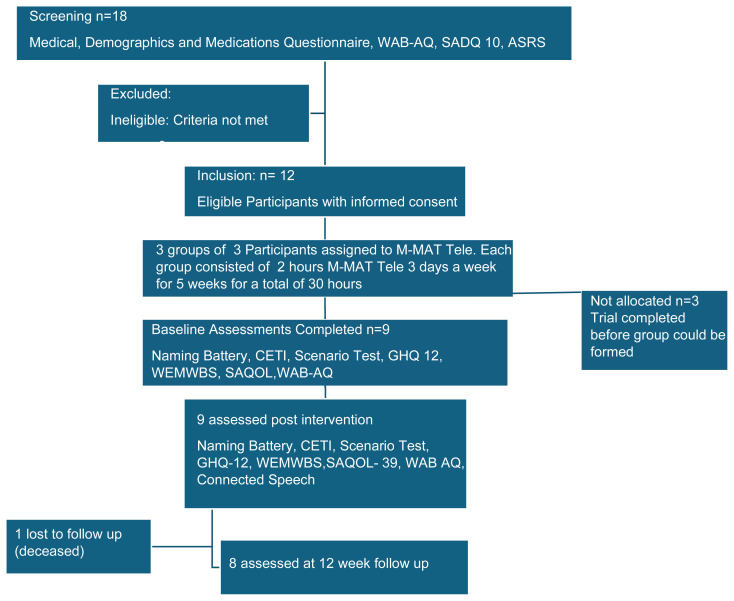
Consort Diagram

Eligible participants needed to be aged 18 years or older, living in Australia, fluent in English prior to their stroke and have chronic aphasia resulting from a stroke (at least 6 months post stroke) with an aphasia quotient of <93.8 on the Western Aphasia Battery Revised Aphasia Quotient (WAB-R-AQ; Kertesz et al., 2007). Participants also needed to be willing to use a tablet or computer capable of videoconference and have high speed internet capability (≥25mbps). Prior experience with tablets/computers was not required, but devices were not supplied to participants for the study. Exclusion criteria were non-stroke neurological diagnoses (head injury, neurosurgery, dementia), severe dysarthria, or severe apraxia of speech as measured on the Apraxia of Speech Rating Scale 3.5 (Duffy et al., 2023). Major clinical depression, as indicated by a score 14 or greater (Dijk et al., 2016) on the Stroke Aphasic Depression Questionnaire-10 (SADQ-10, Sutcliffe & Lincoln, 1998), was flagged during screening and discussed with the participant and their family as to whether (1) professional mental health support was indicated and (2) this could affect involvement or adherence to the study protocol. A total of 18 participants were screened using the Medical, Demographics and Medications Questionnaire, WAB-AQ, SADQ-10, and ASRS. Some were excluded because they did not meet eligibility criteria. Twelve participants were eligible and provided informed consent. Of these, nine were allocated to M-MAT Tele in three groups of three. Each group received two-hour sessions, three times per week, for five weeks, giving a total of 30 hours. Three participants were not allocated because the trial ended before their group could be formed. Baseline assessments were completed for the nine allocated participants and included the Naming Battery, CETI, Scenario Test, GHQ-12, WEMWBS, SAQOL, and WAB-AQ. All nine completed post-intervention assessments, which included the same measures plus connected speech. At follow-up, one participant was lost due to death, leaving eight participants who completed the 12-week follow-up. Figure 1 outlines the recruitment and participant flow.

All trial procedures were conducted over videoconference, meaning there were no restrictions on location within Australia.

Participants were recruited through a variety of advertising methods which included newsletters, social media channels, mailing lists and clinician forums. Participants were grouped according to aphasia severity on the WAB-R AQ (Mild 62.6–93.6, Moderate 31.3–62.5, Severe 0–31.2) and therapy groups scheduled once a group of three was formed. Aphasia friendly information and consent forms were developed to maximise the accessibility of trial participation. Consent was gained via Zoom calls in which the research team was able to confirm that participants understood their involvement in the trial.

### Assessments

Trained, qualified speech pathologists conducted assessments by videoconference using Zoom. In addition to eligibility screening, participants were assessed at three timepoints: baseline, post intervention and 12 week follow up. Table 1 illustrates the outcome measures used to assess various domains and the timepoints in which these were taken. Assessment sessions were video recorded to allow confirmation of scoring where necessary. Each participant was evaluated by the same assessor wherever possible to maximise reliability. Each assessor’s first assessment was reviewed by the research team to rescore and check fidelity of the spontaneous speech section of the Western Aphasia Battery and Apraxia of Speech Rating Scale 3.5. The spontaneous speech section of the WAB-R was also rescored if participants were within three points of the cutoff score of 93.8.

### Intervention

M-MAT Tele was delivered for two hours per day, 3 treatment days per week, over 5 consecutive weeks (30 hours) in line with a previously piloted schedule of in-person M-MAT (Pierce et al., 2023). Therapy was delivered by qualified therapists who were trained in the M-MAT Tele protocol during a 2.5-hour session, provided with an intervention handbook, and offered phone and email support with researchers JEP and EE throughout the M-MAT Tele intervention period.

M-MAT Tele uses card-based games played by participants. Each card has a photograph of the target noun or verb and when it is their “turn” in the game, participants must produce the target word to progress. For example, in the game “memory,” participants must nominate a card to be turned over by producing the target noun or verb on the top side of the card. Target words may be produced in isolation (the starting level for those with severe aphasia), in carrier phrases or short sentences (moderate aphasia starting level), or in longer sentences (mild aphasia starting level). When word retrieval is unsuccessful, a multimodal cueing hierarchy is used, guided by the therapist. The participant is assisted to (1) gesture, (2) draw, (3) read and (4) write the target word while repeating it aloud. M-MAT Tele software has exemplar cues pre-recorded.

A prescribed set (easy, moderate, hard) of coloured pictures is used as therapy stimuli prescribed based on the group’s baseline aphasia severity (mild/moderate/severe). Each set contains five subsets – 3 noun subsets and 2 verb subsets. In total, there are 80 picture stimuli per set, with 16 per subset. Sessions alternate each hour between noun and verb subsets.

Participants within groups have the same aphasia severity; however, fluency, agrammatism, word finding ability, and error patterns are not matched. To accommodate these differences, target phrases and sentences were individualised according to each participant’s performance. Participants progressed through increasingly more challenging linguistic levels based on scores from the previous session to ensure therapy remained sufficiently challenging. There were six linguistic levels for nouns and six for verbs, each with a specific phrase/sentence structure in which to produce the target (see appendix for examples). The therapist initially used written sentence frames, included in the software, to support participants to produce accurate sentences but these were only used where needed by the participants and withdrawn over time.

The first session of each group involved co-creation of group rules to establish rapport and facilitate a supportive environment. This involved discussions around group ambience, expectations, explanations of the treatment, and other housekeeping matters such as scheduled breaks. Each session commenced with a review of any changes to participants’ medications, any adverse events since the previous treatment day as well as discussion about whether home practice had been completed or not.

Participants had a choice of 3–4 digitised games to select from: memory, bingo, guess the card, and snap. Snap was developed during the data collection phase, after the first group, based on early feedback from participants that a greater variety of games was required. Typically, games were changed each hour, or sooner if participants’ interest was waning or if the group agreed. A 15-minute break was provided after the first hour of therapy (noun session) prior to commencement of the second hour (verb session). Therapists shared their screen and controlled the cursor whilst participants directed the therapist verbally to select cards for them. Communication support strategies such as yes and no questioning were employed if required. A TIDIER checklist was used to for M-MAT Tele to improve completeness of reporting and replicability. For further details of the M-MAT Tele software, see Pierce et al (2024).

Following the therapy session, a personalised 15-minute home practice task was assigned to each participant aiming to support transfer of learning to everyday communication. Tasks were based on each participant’s therapy targets and linked to their intended activities after leaving the therapy sessions.

During the trial, participants continued with their usual care. Usual care was not logged as the trial focus was on feasibility and acceptability.

### Outcome Measures

Table 2 outlines the key domains being explored in this trial and the measures for each. Acceptability was considered independently of feasibility, although it is typically considered necessary for overall feasibility in many models (Teresi et al., 2022). Trial and intervention feasibility were also considered independently, as the former considers the feasibility of scaling up the trial and the latter, the feasibility of eventual implementation (e.g., Hubbard et al., 2016).

#### Trial Feasibility

Measures of trial feasibility included the number of inquiries, the eligibility rate (% of inquiries), the recruitment rate (% of target), changes in distress and fatigue self-ratings following assessments, dose delivered (% hours delivered as scheduled), and treatment fidelity. To assess treatment fidelity, all M-MAT Tele sessions were videorecorded. Following each group’s first day, the video was reviewed by the research team to check the therapist’s adherence to the protocol (see Figure 2 for a compliance checklist). As needed, feedback or support was provided to the therapist within 48 hours to ensure adherence to the protocol at the following session. A random quarter (15 mins) of the midpoint therapy session (hour 15 of 30) was also reviewed for compliance using the checklist, with feedback communicated to the therapist. A fidelity rate of 95% compliance was anticipated given past research (Pierce et al., 2023; Rose et al., 2022). Fatigue and distress were assessed using a 0–10 vertical numeric scale with visual anchors at each end, an accessible format previously employed in the COMPARE trial (Rose et al., 2022). The minimum dose delivery threshold was set at 80% in line with the COMPARE trial (Rose et al., 2022).

#### Trial Acceptability

Trial acceptability was explored based on the number of withdrawals and the completion rate for assessments. Withdrawals from M-MAT in the earlier sub-study of in-person intervention at the same schedule were low (1/17; 6%) while the assessment completion rate for all participants was 99% (Pierce et al., 2023).

#### Intervention Acceptability

Intervention acceptability comprised participant responses to a treatment satisfaction questionnaire and attendance rates (proportion attending >80% of prescribed sessions). The aphasia friendly treatment satisfaction questionnaire was adapted from Pitt et al. (2017) and was completed with participants to gain insight into their views and experiences of M-MAT Tele. Questions included perceived effectiveness of M-MAT Tele, convenience and accessibility of online intervention, enjoyment and engagement, technical experience, comfort and preference with online therapy, and overall impressions. The questionnaire used a Likert rating scale and three open-ended questions. Assessors provided communication support to participants to answer open-ended questions and/or add additional comments to Likert scale questions.

#### Intervention Feasibility

Intervention feasibility included the number of technical problems experienced during intervention, the number of adverse events, changes in distress and fatigue ratings during therapy, and the number of software modifications required. In the COMPARE sub-study, participants completed M-MAT on the same schedule as the present trial and reported low symptom levels: median fatigue scores were 1 (IQR 0–2) at the start of a therapy day and 1 (IQR 1–3) at the end, while median distress scores were 0 and 1 at the beginning and end of the day, respectively (Pierce et al., 2023).

### Clinical Outcomes

A range of clinical outcomes likely to be used in future trials were collected and the changes in these were examined. Outcomes were based on previous, in person trials of M-MAT (Rose et al., 2022) with the addition of mood and wellbeing outcomes, which could foreseeably change following an interactive, group intervention. The baseline, post intervention and follow-up quantitative data were examined at both individual and group levels. At the individual level, changes from (a) baseline to post intervention and (b) baseline to 12 week follow up were compared to the Smallest Detectable Change-90 thresholds (Breitenstein et al., 2022). At the group level, standardized mean differences (Cohen’s *d*) were calculated for the same timepoint comparisons (a) and (b). All analyses were conducted in RStudio (2025.05.0 – 496).

#### Therapist Interviews

Semi-structured interviews with two trial therapists provided an additional perspective on trial and intervention acceptability and feasibility. The interviews were conducted within one month of the therapists’ last intervention session and were recorded online over Zoom. The research questions utilised in the interviews were evaluative in nature. They aimed to appraise the suitability and effectiveness of the study processes and intervention (e.g. “Tell us about any technical difficulties you encountered and how they affected the treatment.”; “What changes would you like to see that could make M-MAT Tele better?”). See Appendix F for the topic guide. The interviews were conducted by researcher EE.

The interview transcripts were analysed by two researchers (JP and EE) using Framework Analysis (Parkinson et al., 2016; Ritchie & Spencer, 2002). This analysis approach allowed the researchers to consider the a priori domains of trial feasibility and acceptability and intervention feasibility and acceptability while also considering emergent themes identified in the data. The following processes were completed:

1. Familiarisation: Automated transcription of the interviews was conducted using Microsoft Stream, and transcriptions were checked and re-checked for accuracy against the recordings by both researchers. Separate reading and re-reading of the transcripts facilitated immersion in the data.
2. Coding of the transcript: Independent coding of the two transcripts was completed by both researchers. This was undertaken line by line. The researchers then jointly reviewed the corresponding transcript and codes, reaching a consensus on codes.
3. Indexing the transcript: EE independently generated potential subthemes for each code. Both researchers reviewed the data and came to an agreement on the subtheme for each code. Subthemes were re-reviewed and checked for accuracy and relevance in a second meeting.
4. Mapping and Interpretation: Four predetermined themes (based on the goals of the study: trial feasibility, trial acceptability, intervention feasibility, intervention acceptability) were identified. Together, the researchers worked through the subthemes, assigning them to an appropriate theme within the framework. The interpretation, meaning and scope of each code and subtheme was checked, reviewed and revised to ensure the researchers were in agreement with the theme allocation.

To ensure quality and trustworthiness of the therapist interview data, we followed four criteria (Lincoln & Guba, 1985). Credibility was addressed by allowing a lengthy period for the review and analysis of the qualitative data, plus regular consultation with an experienced qualitative researcher (MC) to guide analysis and processes. Transferability of the data collected was preserved through clear description of the participants interviewed and timing of the interviews. Through keeping detailed digital notes of steps taken during the qualitative analysis, the dependability of the process was maintained. Confirmability of the data was addressed by both researchers referring back to the transcript of the interviews and reviewing the link between the original quote and planned coding and analysis.

## Results

### Participants

Recruitment of nine participants occurred between February – July 2023 and was achieved as planned. The final cohort completed their follow up assessments in January 2024. The median age of all participants was 56 (25.5 IQR) with a range of 28–83 years. Seven participants were male (77.7%) and two participants were female (22.2%). At baseline, six (66.6%) participants had mild aphasia, three (33.3%) had moderate aphasia and no participants had severe aphasia. Participants were a median of 2.2 years (IQR 6.9 years) post-stroke (range 11 months to 32 years). Baseline demographic and characteristics are reported in Table 3.

### Trial Feasibility

There were 26 inquiries for participation in this study. Eighteen consented (69%) and were screened for eligibility, and 12 participants met the inclusion/exclusion criteria (66%). Three of these 12 participants were unable to be scheduled into a group before the conclusion of the trial as there were insufficient participants of the same severity to form a group of three.

Mean distress ratings increased from 1.9 to 3.4 following screening assessments (n=14) and 1.9 to 3.0 during outcome assessments (n=21). Mean fatigue ratings increased from 2.8 to 3.5 following screening assessments and 2.0 to 2.8 for outcome assessments. Both values were higher than the ratings recorded before and after intervention sessions (see below). Previous trials of M-MAT have not reported fatigue and distress ratings associated with assessment sessions.

The mean duration of therapy sessions was 57.40 minutes for noun practice and 54.86 minutes for verb practice, both close to the target of 60 minutes. Therapy integrity was high: fidelity checks were completed as planned, with a mean adherence rate of 94% across sessions, comparable to the 95% compliance anticipated from previous research.

### Trial Acceptability

There were no withdrawals from the trial. All recruited participants completed baseline and post intervention assessments, and 8/9 participants completed 12 week follow up assessments. One participant died during the follow-up period. In total, assessment completion rates were 183/198 (92.4%).

### Intervention Acceptability

A median of 24.4 hours (1464 minutes) or 81% of the prescribed dose of M-MAT Tele was provided with a median daily therapy time of 1.9 hours (115 minutes of target 120). Six participants attended more than 80% of the prescribed treatment days. The remaining participants completed 73.3% (n=1), 66.6% (n=1) and 60% (n=1) of the prescribed days. The main reasons for not attending sessions were therapist and participant illness (including COVID-19 and a new major psychiatric diagnosis), medical appointments and prearranged travel or work. One missed session was noted as having been forgotten and on one occasion no reason was recorded. A median of 13 therapy days was delivered per participant. Participants with moderate aphasia attended 38/45 (84%) of sessions and those with mild aphasia, 74/90 (82%). This attendance rate and thus median hours delivered was lower than expected compared to previous, larger studies of in-person M-MAT. In the COMPARE trial, the median M-MAT hours received was 29.9 for COMPARE (Rose et al., 2022) and 29.5 in the sub-study (Pierce et al., 2024). The median of 24.4 hours in this trial is nonetheless above the suggested minimum of 80% (24 hours) from Rose et al. (2019).

Table 4 contains a summary of all satisfaction questionnaire responses for M-MAT Tele. Overall, eight participants rated M-MAT Tele as positive or very positive; one participant rated the intervention as neutral. Seven of nine participants indicated they would “definitely” recommend M-MAT Tele to others. One participant rated most elements as neutral and did not elaborate during opportunities for open ended feedback (P4). Feedback on the practical aspects of M-MAT Tele were very positive, with no issues reported in seeing or hearing the clinician, and minimal assistance required to access Zoom/M-MAT Tele. Other responses to the open-ended items of the satisfaction questionnaire reflected both positive feedback and constructive suggestions for improvement. Four participants described increased confidence in daily communication and the ability to produce longer sentences, while two were unsure whether they had improved substantially. Feedback on the practical aspects of M-MAT Tele were very positive, with no issues reported in seeing or hearing the clinician, and minimal assistance required to access Zoom/M-MAT Tele. Instances of technical issues were minimal, and participants commented on the overall ease of use (e.g., “I think the way the whole thing’s set up is good.” P6). The convenience and accessibility of M-MAT Tele was viewed as positive by participants, including the ability for people from different areas to attend and avoid travel time, traffic, and accommodation costs (“In our group we had one guy from Perth, one in Victoria and me, NSW… it’s just great.” P1; “Treatment people are not there in [name of town]” (*i.e., there are no clinicians available where I live*) P9). One participant had previously completed in-person M-MAT and reported they preferred the telerehabilitation medium as they avoided driving and traffic. Receiving treatment in a group was consistently highlighted as positive, with comments about making new friends, inspiring one another and feeling less alone (“Come to this session and have a chat, and do therapy as well.” P1; “A friend, new one. And he’s amazing, he was a stroke as well.” P5; “my speech pathologist is on a one-to-one sort of therapy, so having a larger group is very motivating because I can see that I’m not alone. … It inspired me to work and improve myself harder.” P7). Two participants with milder aphasia, who achieved the highest grammatical level during treatment, critiqued the level of challenge within the therapy levels, stating a preference for more challenging targets (“but I think that there’s not much on offer for my degree of aphasia.” P2; “Include as much as possible because there are times that it is really getting redundant” P7). Finally, the M-MAT Tele schedule was acceptable, with several comments that the dose and duration were appropriate (“five weeks was the right amount of time.” P2; “Three times a week was good. Any more than that would be probably too much… Anything longer than two hours, for me, would probably be too long” P6).

### Intervention Feasibility

Software modifications were implemented during and following the intervention phases based on feedback, which included larger sized items to account for visual challenges and formatting changes to improve accessibility for participants with colour blindness. Based on observations of session recordings, therapists were asked to maximise exposure to therapy tasks by engaging other participants when it was not their turn in the cueing hierarchy as some participants appeared disengaged whilst awaiting their turn. Protocol changes suggested by therapists included adding an additional linguistic level to both verbs and nouns for subsequent trials to provide greater challenges for milder participants who reached the highest level well before the intervention was completed. Minor changes were implemented immediately whilst other changes such as the adding additional linguistic levels are planned for future trials.

Technical errors were noted for two sessions. In the first, a program issue in the M-MAT Tele software meant that the therapist could not select verbs for session two and had to complete two noun sessions. This was fixed for the subsequent session. The second error related to temporary internet dropouts during therapy.

A total of either eight adverse events were recorded during the trial (i.e., one serious, five moderate and two mild), with seven of these deemed unrelated. One moderate event was deemed as possibly related to exacerbation of fatigue which resulted in reduced attendance. This instance was recorded as possibly trial related as it was unclear whether this fatigue was secondary to the trial or due to a new medical diagnosis. The serious adverse event was one death (unrelated to the study), occurring prior to the 12 week follow up timepoint.

During intervention, the mean distress rating was 1.1 at the beginning of the day and 1.6 at the end of the day; comparable to the 0 and 1 median ratings in a previous trial of the same schedule (Pierce et al., 2023). Mean participant fatigue increased from 1.3 to 1.85 within each day, again equivalent to the prior trial (Pierce et al., 2023). Mean therapist fatigue was unchanged before and after each day, at 1.4 (one of three therapist’s fatigue scores was unavailable due to a database error). No prior therapist fatigue ratings are available for comparison.

As shown in Table 4, participants generally reported positive experiences with M-MAT Tele. Most felt their communication had improved, with three saying “definitely yes,” two “yes,” two neutral, and two “no.” Similarly, most said they had gained new skills, with four “definitely yes” and three “yes,” while a smaller number were neutral or disagreed. Nearly all participants agreed that online therapy was a good way to receive treatment and that it helped them save travel time, with seven “definitely yes” responses in each case. When asked about the therapy content, most enjoyed the games and found the cues easy to follow. The majority also felt the program ran smoothly. In terms of expectations, four participants felt the program was “definitely as good” as they thought it would be, while two were positive and three were neutral. Seven participants said they would recommend M-MAT Tele to others. Finally, when asked for an overall rating, the majority responded positively: five “very positive,” three “positive,” one neutral, and only one negative.

### Clinical Outcomes

Treatment outcomes at immediately post intervention and 12 week follow up are presented in Table 5 for group level results and Table 6 for individual results. Large effect sizes were seen on word retrieval of treated items (1.952), communicative quality of life (1.196) and functional communication (1.047) post intervention. Medium effect sizes were observed for word retrieval of untreated items, wellbeing and multimodal communication. At the 12 week follow up assessment, word retrieval maintained a very large effect size (1.431) along with multimodal communication (large - 0.8). The individual outcomes demonstrate variable results between participants, though improvements on communicative quality of life were more consistent.

### Therapist Interview Outcomes

Two of the three therapists were interviewed. Both therapists conducted M-MAT Tele intervention for one group each, with one therapist in a job share role conducting 2/3 sessions each week. The third therapist did not respond to requests to participate in a semi-structured interview.

#### Trial Feasibility

Therapists reported that involvement in the trial was well supported by the responsive research team, the therapy training, and trial procedures. *‘Whoever's designed and set up all this stuff with it, pre-record in all the meetings… they’ve done a fantastic job because it's really made me not have to think about the technical side’* (SP1). However, one therapist noted that participation in the study did require a certain level of skill with technology ‘*I suppose it's assumed knowledge that you know how to work a computer…. I couldn't help [a participant with a technical issue]’* (SP1). They identified that additional orientation and training in using telerehabilitation for delivering intervention would be helpful.

#### Trial Acceptability

Acceptability of the trial was confirmed by both therapists, and materials such as data collection forms were ‘*really easy to use’* (SP2). It was suggested that other procedures for therapists could have been streamlined to reduce time spent completing repetitive administration tasks *‘I asked if it was possible to have some of the forms auto populate.’* (SP2). Job sharing was framed as a positive component of the trial – for both therapists and as a way of increasing variety for participants: *‘It actually worked really well though, to be honest. Like the job share…It definitely had some benefits, just in terms of, I suppose that sort of different therapist and a bit of “fresh meat” for them [the participants], if you know what I mean.’*(SP1).

#### Intervention Feasibility

Providing the M-MAT Tele intervention was considered feasible by the two therapists, with both describing that the telerehabilitation platform and M-MAT Tele software largely worked as anticipated: *‘And the things that were challenges were mostly just glitches that we've addressed as they've come up... But I think the software itself, the idea of how it's meant to work all pretty much makes sense’* (SP2). Both therapists were forthcoming in suggesting changes to the software, such as improving the size and clarity of the stimuli used: ‘*some of the pictures ... were on, like a whiter background. That was a bit of an issue sometimes because you couldn't… they couldn't distinguish what the picture was’* (SP1); *‘I couldn't really distinguish whether it was a visual issue or an aphasic issue. I think if you could enlarge the size of that picture* ...*’* (SP1). One therapist described in detail the need to consider the role of carers in the intervention including what instructions are given to them at the start of therapy ‘*It will depend a lot, I think, on the dynamics of the partners and the people in the room… Probably shouldn't be a blanket rule one way or the other, but it's just something to think about. Because it may be that if you weren't to say anything, the partners would stay all the way through, and it might actually be preferable to encourage them to give some space.’* (SP2).

#### Intervention Acceptability

Overall, M-MAT Tele intervention was considered to be acceptable: ‘*Definitely enjoyed it, definitely recommend it’* (SP1); and *‘the summary statement is I loved it’* (SP2). Therapists highlighted the specific benefits of the multimodal approach: *‘But I just think communication is inherently multimodal. It's naturally multimodal. We all use gestures and pointing and facial expressions and sound effects. And we'll often use writing or referring to writing’ (*SP2). A particularly positive aspect of M-MAT Tele was that participants enjoyed the opportunity to work in a group: *‘And they all share their knowledge and experiences that I don't know anything about. So they can, you know, bond over that and share in that. And they teach me stuff, rather than it just being me giving them therapy. So I think that's an inherently really beautiful thing about working in groups.’* (SP2). However, they also reflected that group dynamics and differences in participants could potentially create challenges in future groups. The previous knowledge and skills of the therapist was also considered important for delivering the therapy *‘But for me, already being familiar with the therapy, transitioning it to telehealth was really quite smooth... I do have a lot of experience in just using Zoom in general’* (SP2). Conversely, it was also viewed as a unique opportunity to develop experience and competency in a new area of clinical practice, *‘I think it's definitely developed my own skills in terms of delivering group therapy’* (SP1).

## Discussion

This pilot study presents initial data on M-MAT Tele, a telerehabilitation adaptation of the evidence-based, group-based M-MAT intervention, co-designed with end users (Pierce et al., 2024). Overall, the findings indicate that the M-MAT Tele intervention is both acceptable and feasible to participants and therapists. All suggestions for procedural and software modifications were minor and easily implementable. Further evaluation of M-MAT Tele in subsequent trial phases is warranted as eligibility and consent rates were high, fatigue and distress rates remained low, and M-MAT Tele was provided with high fidelity. In addition, there were no withdrawals and the majority of assessments were completed as planned. Preliminary clinical outcomes suggest potential for therapeutic benefit, supporting further investigation.

In terms of feasibility, this trial was run entirely online, including trial personnel training, consenting participants, assessments, and therapy delivery. There were no major technical issues relating to videoconference for consent, assessments, or therapy. These outcomes also affirm that efforts to design accessible software and procedures were successful. Notably, no internet dropouts occurred, in contrast to an earlier group telerehabilitation study (Pitt et al., 2017), which reported multiple dropouts during intervention. This may reflect improved connectivity in Australia since completion of the National Broadband Network (NBN, 2023). Online assessment completion rates were high, further supporting the feasibility of remote assessment. A limitation of this study is that not all assessments have been specifically validated to be delivered online, a pressing need as telerehabilitation becomes increasingly routine (Hilari et al., 2024).

Overall, M-MAT Tele was deemed acceptable and received positive feedback from participants and therapists. In particular, both appreciated the group environment and connecting with others with aphasia, findings in line with other group-based telerehabilitation studies who report high satisfaction levels (Pitt et al., 2019). However, feedback also suggested that acceptability may vary according to aphasia severity. For example, two participants with mild aphasia expressed dissatisfaction with the inclusion of drawing and the structured cueing hierarchy. M-MAT was designed to treat a wide range of aphasia severities and presentations, but some treatment components may be more beneficial for individuals with more severe aphasia. Furthermore, participants with milder aphasia noted that the final linguistic level of M-MAT Tele lacked sufficient challenge, indicating a potential challenge ceiling. Given the limited availability of interventions tailored for mild aphasia (Mozeiko & Pascariello, 2020), modifications are planned to further tailor M-MAT for people with mild aphasia.

The intervention schedule, which is less intensive than the original M-MAT, also received positive feedback, with several comments that longer sessions, more days per week or a longer duration would be too demanding. While high-intensity aphasia interventions are supported by evidence, little is known about the preferences of people living with aphasia regarding scheduling (Pierce et al., 2020), and these comments suggest that a more distributed schedule could be preferable for some.

Therapy attendance rates varied across participants. Despite this variability, the median total therapy time still exceeded the minimum 24 hours (80%), indicating that participants were engaged overall. Unlike individual therapy, group-based sessions offer limited flexibility for rescheduling, which may further impact attendance. Notably, more than half of all missed sessions were due to COVID-19 or other illnesses. Other contributing factors impacting attendance rates included therapist illness, specialist medical appointments that could not be rescheduled, and prearranged travel or work. Taken together, these data suggest that most absences were due to extenuating circumstances rather than factors relating to the intervention. Nonetheless, further investigation is needed to fully assess the acceptability of M-MAT Tele compared to in-person M-MAT, and particularly the differing attendance rates.

On balance, despite some calls for increased challenge and variable attendance, participants reported broadly positive experiences with the modality of therapy delivery, the software, the group environment, and the schedule. These metrics and feedback will inform future studies of M-MAT Tele, particularly in relation to the protocol modifications and modality.

While the primary aim of this pilot study was to assess feasibility and acceptability, preliminary clinical outcomes were also explored. Given the small sample size (n=9), these findings must be interpreted with caution and are not intended to demonstrate efficacy. However, they provide an early indication of the potential therapeutic value of M-MAT Tele and offer guidance for outcome selection and measurement in future, larger-scale trials. M-MAT tele led to improvements immediately post-therapy at the group level in most areas, with particularly strong group-level effect sizes in word retrieval, functional communication, and communication-related quality of life. These findings align with those of the COMPARE trial, which also found significant improvements in word retrieval, functional communication and communicative quality of life following M-MAT compared to the usual care arm (Rose et al., 2022). Additionally, two participants demonstrated improvements in their mood compared to baseline. Mood has not previously evaluated in M-MAT trials; we hypothesised that making gains in language within a group setting may yield positive emotional effects in participants.

One of the challenges encountered in this pilot trial was recruiting individuals with severe aphasia. We used accessible recruitment and consent materials but did not have sufficient participants to form a group. Similarly, only 2.7% of the COMPARE trial sample were classified as having severe aphasia (WAB AQ <31.3), despite the trial criteria including all severities. This may indicate a limitation in successfully reaching people with severe aphasia through our recruitment methods or reduced confidence of people with severe aphasia to participate in aphasia intervention. Regardless, the result is that we do not yet have data to establish whether this treatment is feasible and acceptable for this sub-population.

Overall, this pilot study provides evidence that both the M-MAT Tele intervention and the trial procedures are acceptable and feasible. Participants and clinicians responded positively to the telerehabilitation format, group-based delivery, and adapted materials, while recruitment, retention, and assessment protocols were successfully implemented. Although preliminary, the clinical outcomes suggest potential consistency with the COMPARE trial (Rose et al., 2022). These findings will inform refinements to M-MAT Tele and offer important guidance for future Phase II exploratory trials.

## Figures and Tables

**Table 1:** Assessment Schedule

Domain	Outcome measure	Screening	T1: Baseline	T2: Post	T3: 12w follow-up
Fatigue & distress	Fatigue and distress rating scales	✓	✓	✓	✓
Medical	Medical history, active medications (Medication changes reviewed each session).	✓			
Apraxia & Dysarthria	Apraxia of Speech Rating Scale	✓			
Aphasia Severity	Western Aphasia Battery (WAB)	✓	✓	✓	✓
Word Retrieval	Naming Battery		✓	✓	✓
Functional Communication	Communication Effectiveness Index (CETI)		✓	✓	✓
Multimodal Communication	Scenario Test		✓	✓	✓
Mood	Community Stroke Aphasia Depression Questionnaire-10 (SADQ-10)	✓			
	General Health Questionnaire 12 (GHQ-12)		✓	✓	✓
Psychological wellbeing	Warwick-Edinburgh Mental Well-being Scale (WEMWBS)		✓	✓	✓
Quality of Life	Stroke and Aphasia Quality of Life scale (SAQOL-39g)		✓	✓	✓
Usability and acceptability	Satisfaction questionnaire Therapist semi-structured interview			✓	

**Table 2:** Measures Mapped to Outcome Domains

Trial Feasibility	Trial Acceptability	Intervention Acceptability	Intervention Feasibility	Preliminary Efficacy
– Inquiries – Eligibility and consent rates – Distress and fatigue (assessment) – Dose delivered – Treatment fidelity – Therapist interviews	– Withdrawals – Assessment completion/missing data – Therapist interviews	– Satisfaction questionnaire – Attendance – Therapist interviews	– Software modifications – Technical problems – Adverse events – Distress and fatigue – Therapist interviews	– Clinical outcomes (signal only, given sample size and design)

**Table 3:** Baseline Characteristics

**Age (Years)**	55 (Mean)
	15.5 (SD)

**Education**	20 years (Median)
	6.5 years (IQR)
	Range 27 to 81

**Sex**	
Male	(7) 77.7%
Female	(2) 22.2%

**Time post onset**	
<12 months	1
13 months- 4 years	5
>5 years	3

**WAB -AQ**	
Mild (62.6–93.6)	6 (66.6%)
Moderate (31.3–62.5)	3 (33.33%)
Severe (0–31.2)	0 (0%)

**Aphasia subtypes**	
Broca’s	3
Conduction	2
Anomic	4

**Apraxia severity (ASRS 3.5) TOTAL Score**	
0–5 (absent)	4 (44.4%)
6–8 (likely absent)	0 (0%)
9–14 (mild)	3 (33.3%)
15–23 (moderate)	2 (22.2%

**Dysarthria severity**	
Absent	(7) 77.7%
Mild	(2) 22.2%

**Stroke type**	
Haemorrhagic	(4) 44.4%
Ischaemic	(5) 55.5%

**Location**	
Metro	(6) 66.6%
Rural	(3) 33.3%
Remote	(0)

**Group demographics (age)**	
Group 1 (moderate)	61–81y
Group 2 (mild)	44–57y
Group 3 (mild)	27–71y

**Group demographics (education level)**	
Group 1 (moderate)	16–21y
Group 2 (mild)	12–24y
Group 3 (mild)	16–28y

**Group demographics (sex)**	
Group 1 (moderate)	3M
Group 2 (mild)	2F, 1M
Group 3 (mild)	3M

**Group demographic (time post stroke)**	
Group 1 (moderate)	1–5y
Group 2 (mild)	1–32y
Group 3 (mild)	10 months – 2y

**Group demographics (aphasia type)**	
Group 1 (moderate)	Broca’s | Conduction | Wernicke’s
Group 2 (mild)	Conduction | Conduction | Wernicke’s
Group 3 (mild)	Broca’s | Anomic | Anomic

**Table 4:** Summary of M-MAT Tele Participant Feedback Questionnaire

	No, definitely not	No, I don't think so	Neutral	Yes, I think so	Yes, definitely so
Has your communication improved since being in M-MAT Tele		2	2	2	3
		●●	●●	●●	●●●
Have you gained new skills from participating in M-MAT Tele		1	3	1	4
		●	●●●	●	●●●●
Could you easily see the speech pathologist?				2	7
				●●	●●●●●●●
Could you easily hear the speech pathologist				1	7
				●	●●●●●●
Did you feel comfortable receiving therapy online?			1		8
			●		●●●●●●●
Did you find having therapy at home was easier?			1	2	6
			●	●●	●●●●●●
Do you think having therapy online is a good way to receive treatment?			1	1	7
			●	●	●●●●●●●
Did you save travel time during M-MAT Tele?			1	1	7
			●	●	●●●●●●●
Did you enjoy the games?			2	3	4
			●	●●●	●●●●
Were the cues easy to follow?			1	3	5
			●	●●●	●●●●●
Did M-MAT Tele run smoothly?			1	2	6
			●	●●	●●●●●●
Was M-MAT Tele as good as you thought it would be?			3	2	4
			●●●	●●	●●●●
Would you recommend M-MAT Tele to others.		1	1		7
		●	●		●●●●●●●
	Very negative	Negative	;Neutral	Positive	Very positive
Overall, what do you think of M-MAT Tele?			1	3	5
			●	●●●	●●●●●

**Table 5:** Group-level Outcomes at Post Intervention and at 12 Week Follow Up

Outcome		Baseline	Post Intervention	12 Week Follow Up	Cohen’s d	

					Baseline–Post	Baseline-Follow up
Naming Battery	Treated	41.7 (19.9)	55.3 (21.7)	48.4 (26.3)	**1.952**	**1.431**
	Untreated	63.9 (28.8)	67.8 (25.9)	67.1 (28.2)	0.754	0.402
SAQOL-39g (communication subscale)		2.86 (0.98)	3.40 (1.18)	3.48 (1.1	**1.196**	0.72
CETI		56.3 (22.5)	61.1 (19.6)	58.8 (14.4)	**1.047**	-0.202
WAB-AQ		70.1 (19.2)	72.0 (20.2)	73.3 (21.1)	0.468	0.54
WEMWBS (wellbeing)		50.6 (11.0)	52.9 (11.6)	55.1 (12.3)	0.583	0.387
GHQ-12 (-ve mood)*		*12 (6.5 - 17.5)*	*6 (-2.5 - 14.5)*	*9.50 (3 - 16)*	*−0.018*	*0.104*
Scenario Test*		*48 (36 - 60)*	*52 (39 - 65)*	*52 (46.75 - 57.25)*	*0.512*	***0.8***

*Note*: 0 – 0.19 Negligible, 0.20 – 0.49 Small, 0.50 – 0.79 Medium, ≥ 0.80 Large, −0.2 – 0 Small negative

* Presented as Median and interquartile range.

**Table 6:** Individual Outcomes

Participant	Baseline	Post	Follow Up	Baseline-Post change	Baseline-Follow up change
Western Aphasia Battery Revised AQ - SDC90 8.26					
P1	74.4	83.6	84.2	9.2	9.8
P2	87.6	95	92.8	7.4	5.2
P3	51.6	56	59.6	4.4	8
P4	45	45.2	49.2	0.2	4.2
P5	64.4	67.2	-	2.8	-
P6	92.8	90.6	94.4	-2.2	1.6
P7	95.6	94.8	96	-0.8	0.4
P8	50.4	44.8	44.2	-5.6	-6.2
P9	66.6	71.2	65.8	4.6	-0.8

Naming battery – treated items (total out of 80). No SDC_90_ available.					
P1	68	79	79	11	11
P2	68	78	74	10	6
P3	18	40	48	22	30
P4	9	7	12	-2	3
P5	41	54	-	13	-
P6	39	56	51	17	12
P7	62	78	75	16	13
P8	31	48	37	17	6
P9	39	58	60	19	21

Naming battery – untreated items (total out of 100). No SDC_90_ available.					
P1	93	95	95	2	2
P2	88	96	96	8	8
P3	31	39	54	8	23
P4	9	17	9	8	0
P5	67	67	-	0	-
P6	75	75	76	0	1
P7	93	93	89	0	-4
P8	38	50	43	12	5
P9	81	78	75	-3	-6

CETI – SDC90 11.53					
P1	75.81	75.13	75.63	-0.69	-0.19
P2	68.19		48.31		-19.88
P3	48.44	66.38	55.13	17.94	6.69
P4	28.75	40.25	44.06	11.50	15.31
P5	-	56.19	-	-	-
P6	83.31	91.69	77.88	8.38	-5.44
P7	-	-	-	-	-
P8	63.63	62.94	51.75	-0.69	-11.88
P9	26.06	35.13		9.06	

SCENARIO TEST – SDC90 4.57					
P1	50	54	53	4	3
P2	54	54	54	0	0
P3	41	36	51	-5	10
P4	31	41	45	10	14
P5	48	48		0	
P6	54	54	54	0	0
P7	53	54	54	1	1
P8	32	35	32	3	0
P9	44	52	50	8	6

SAQOL-39g (Communication subscale) – SDC90 0.4					
P1	4.71	4.86	4.29	0.15	-0.42
P2	2.86	3.71	3.29	0.85	0.43
P3	2.86	3.86	4.43	1.00	1.57
P4	1.86	2.00	2.14	0.14	0.28
P5	1.57	2.14		0.57	
P6	3.71	4.71	4.71	1.00	1.00
P7	3.29	4.14	4.14	0.85	0.85
P8	2.00	1.71	2.00	-0.29	0.00
P9	2.86	3.43	2.86	0.57	0.00

GHQ-12 – SDC90 4.06					
P1	0	2	3	2	3
P2	10	6	10	-4	0
P3	2	2	0	0	-2
P4	15	30	23	15	8
P5	22	-	-	-	-
P6	12	6	8	-6	-4
P7	12	2	9	-10	-3
P8	13	15	14	2	1
P9	-	-	13	-	-

*Note*. Green = exceeded SDC_90_, Red = exceeded SDC_90_ in negative direction

## Appendix A Game explanations

Memory involved finding matching shapes that were hidden beneath the naming pictures. Participants ask the therapist to turn over two cards by producing each target in a carrier sentence. If a matching pair of shapes is not found, the participant is encouraged to say, “I don’t want a red heart”. If the pair is found, a participant might say, “I have 2 red hearts”. Bingo involved checking off all the all shapes on their named bingo card. Participants were given three random shapes on their named ‘card’. Participants took turns to select a card from the middle board by verbally producing the items. The shape beneath the card should be ‘claimed’ by any player who has it on their card, e.g. “I have a green ring.” The clinician needed to check off the shape on matching boards before the game continues.

Guess the card involved participants asking the clinician to place tokens on two pictures by producing each item in the carrier phrase. When all participants have placed their tokens, the clinician pressed the “go” button. The game flashed over the pictures at random and if it ‘lands’ on a picture that has a token on it, that participant will be awarded points. Gold token = 2 points and silver token = 1 point. Tokens were reset after each turn to ensure plenty of language practice. The first person to ten points wins.

Snap was the less favourable game and involved the clinician “turning over” the card of the person whose turn it is by hovering the mouse. The item is produced in a carrier phrase. If the turned over card matches the card in the middle pile, participants can say “snap” to be awarded a point. The clinician should judge who said snap first and manually award them a point. Therapy stimuli was selected to match group severity and did not change during intervention (see appendix) however subset changed when at least two participants scores >80% in one session or nine sessions have passed on that subset. Successful and unsuccessful attempts were recorded on a therapist intervention record sheet and entered into REDcap post sessions. When a participant was unable to retrieve the target word in a reasonable time (approximately 10–20 seconds, or produces another incorrect word for the target a cueing hierarchy is implemented (see appendix). M-MAT Tele does contain a cue menu with examples for a gesture, drawing and the written word of each target. These can help provide examples but did not have to be used.

## Appendix B Therapy Stimuli

**Table:**

Easy set Severe aphasia	Moderate set Moderate aphasia	Hard set Mild aphasia

Nouns A	Nouns A	Nouns A
bed, car, coffee, doctor, dog, hospital, house, key, knife, money, office, table, teeth, telephone, train, water	cake, candle, cereal, computer, couch, dress, flag, glasses, lawnmower, lion, potato, seal, shoe, shower, taxi, toilet	ambulance, banana, celery, chisel, computer, couch, hammock, jam, kettle, menu, microphone, photo, rhinoceros, telescope, toilet, tomato
Nouns B	Nouns B	Nouns B
beach, beer, bread, brush, bus, camera, chair, chicken, fish, garden, meat, milk, piano, roof, tongue, wine	ankle, apple, beer, boot, cheese, cherry, chocolate, dentist, drum, grandfather, hamburger, hammer, pasta, salad, stamp, umbrella	ankle, apple, boot, cheese, cherry, chocolate, cream, dentist, drum, grandfather, hamburger, hammer, mushroom, salad, stamp, umbrella
Nouns C	Nouns C	Nouns C
bath, bird, butter, café, cat, fence, ham, iPad, jacket, nurse, plug, sheep, shirt, soap, tea, toast	bath, bird, butter, café, cat, fence, ham, Ipad, jacket, nurse, plug, sheep, shirt, soap, tea, toast	asparagus, biscuit, caterpillar, dolphin, gloves, grasshopper, jeans, jumper, mug, peacock, scissors, screwdriver, snowman, tongs, volcano, wheelbarrow

Verbs D	Verbs D	Verbs D
building, carrying, cutting, dancing, drinking, driving, giving, helping, looking, opening, playing, reading, running, walking, washing, writing	blowing, breaking, carrying, cutting, drinking, driving, leaning, marching, melting, opening, playing, stretching, sweeping, swinging, walking, washing	arguing, biting, carving, crawling, dipping, folding, frying, kneeling, leaning, marching, proposing, pulling, sinking, sweating, sweeping, watering
Verbs E	Verbs E	Verbs E
camping, cleaning, cooking, crying, drawing, dreaming, drying, eating, falling, raining, sailing, shopping, sleeping, stirring, swimming, typing	bouncing, camping, catching, dreaming, drilling, dripping, drying, floating, hugging, massaging, peeling, raining, sailing, stirring, typing, winking	bouncing, catching, drilling, dripping, floating, hugging, juggling, ordering, peeling, relaxing, skating, sneezing, snowing, tickling, winking, yawning

## Appendix D Linguistic Levels

### Nouns

**Table:**

Level	Grammatical Form	Example
1	Noun Severe aphasia starts here	“Couch?”
2	Carrier phrase + noun Moderate aphasia starts here	“I’d like the couch?” or “I’ll have coffee”
3	Carrier phrase + adj + noun Mild aphasia starts here	“I’d like the red couch?” or “I’ll have a hot coffee”
4	Carrier phrase + adj + adj + noun	“I’d like a large, red couch?” or “I’ll have a nice, hot coffee”
5	Sub + to be + adj + adj + noun	“The girl is on the large, red couch?” or “The man has a nice, hot coffee”
6	Sub + verb + adj + adj + noun	“The girl is sitting on the large, red couch?” or “The man is drinking a nice, hot coffee”

### Verbs

**Table:**

Level	Grammatical Form	Example
1	Verb Severe aphasia starts here	“Sweeping?”
2	Carrier phrase + verb Moderate aphasia starts here	“I’d like ‘sweeping’?” or “I choose sweeping”
3	Sub + verb Mild aphasia starts here	“The girl is sweeping?”
4	Sub + verb + object	“The girl is sweeping the floor?”
5	Sub + verb + object + prep phrase	“The girl is sweeping the floor outside” or “The girl is sweeping the floor with a broom”
6	Sub + verb + object + prep phrase + conjunction SV	“The girl is sweeping the floor with the broom because it is dirty” or “The lady is dancing with her partner in the ballroom because it’s a competition”

## Appendix F Semi-structured interview questions with Speech Pathologist

Please tell us about your experience providing M-MAT Tele

- Prompt: Can you tell us about any benefits for you as a therapist?
- Prompt: Can you tell us about any drawbacks for you?

Please tell us about any technical difficulties you encountered and how they affected the treatment

- Prompt: Were there limitations in:
- Software
- Equipment
- Therapy procedures

Are there any other frustrating moments or experiences you’d like to share?

What changes you would like to see made?

- What could make M-MAT Tele better?
- How might we improve the study forms or procedures for next time?

Would you provide this treatment again in future?

Overall, how would you evaluate your experiences?

Is there anything we haven’t talked about that is important for us to hear?
